# The neurosurgical benefit of contactless *in vivo* optical coherence tomography regarding residual tumor detection: A clinical study

**DOI:** 10.3389/fonc.2023.1151149

**Published:** 2023-04-13

**Authors:** Patrick Kuppler, Paul Strenge, Birgit Lange, Sonja Spahr-Hess, Wolfgang Draxinger, Christian Hagel, Dirk Theisen-Kunde, Ralf Brinkmann, Robert Huber, Volker Tronnier, Matteo Mario Bonsanto

**Affiliations:** ^1^ Department of Neurosurgery, University Medical Center Schleswig-Holstein, Luebeck, Germany; ^2^ Medical Laser Center Luebeck, Luebeck, Germany; ^3^ Institute of Biomedical Optics, University of Luebeck, Luebeck, Germany; ^4^ Institute of Neuropathology, University Medical Center Hamburg-Eppendorf, Hamburg, Germany

**Keywords:** optical coherance tomography, brain tumor imaging, residual tumor detection, tumor border detection, tissue classification, visual image analysis, artificial intelligence, automated tissue characterization

## Abstract

**Purpose:**

In brain tumor surgery, it is crucial to achieve complete tumor resection while conserving adjacent noncancerous brain tissue. Several groups have demonstrated that optical coherence tomography (OCT) has the potential of identifying tumorous brain tissue. However, there is little evidence on human *in vivo* application of this technology, especially regarding applicability and accuracy of residual tumor detection (RTD). In this study, we execute a systematic analysis of a microscope integrated OCT-system for this purpose.

**Experimental design:**

Multiple 3-dimensional *in vivo* OCT-scans were taken at protocol-defined sites at the resection edge in 21 brain tumor patients. The system was evaluated for its intraoperative applicability. Tissue biopsies were obtained at these locations, labeled by a neuropathologist and used as ground truth for further analysis. OCT-scans were visually assessed with a qualitative classifier, optical OCT-properties were obtained and two artificial intelligence (AI)-assisted methods were used for automated scan classification. All approaches were investigated for accuracy of RTD and compared to common techniques.

**Results:**

Visual OCT-scan classification correlated well with histopathological findings. Classification with measured OCT image-properties achieved a balanced accuracy of 85%. A neuronal network approach for scan feature recognition achieved 82% and an auto-encoder approach 85% balanced accuracy. Overall applicability showed need for improvement.

**Conclusion:**

Contactless *in vivo* OCT scanning has shown to achieve high values of accuracy for RTD, supporting what has well been described for ex vivo OCT brain tumor scanning, complementing current intraoperative techniques and even exceeding them in accuracy, while not yet in applicability.

## 1 Introduction

Neurosurgical tumor resection is a crucial part of neurooncological treatment concepts of brain tumor patients and maximizing the extent of tumor resection has shown to have beneficial prognostic impact on overall and disease free survival (1–3). Gross total resection (GTR) of the tumor depends on the surgeon’s experience and skill, as much as the use of intraoperative tools that help differentiate tumorous from healthy brain tissue. Fluorescence-guided surgery after preoperative administration of fluorescent dye, such as 5-aminolevulinic acid (5-ALA) or fluorescein sodium (FNa), has shown to increase the extent of tumor resection and subsequently the progression free survival in glioma patients (4). However, photosensitivity reactions are acknowledged side effects of 5-ALA and adverse reactions, though rare, have been reported for FNa guided surgery. Intraoperative magnetic resonance imaging (MRI) has shown great potential for this purpose, but poses economic challenges to any department, requires specific conditions in the operating room and has shown to significantly increase operative time in comparison to traditional operating rooms (5). Due to factors like intraoperative brain shift, the use of neuronavigation for the detection of tumor borders should always be critically questioned by the operating surgeon and intraoperative ultrasound has not yet shown to increase the extent of tumor resection (6). Improving intraoperative RTD is therefore a crucial part of neurosurgical research and a variety of *in vivo* imaging techniques, such as Raman spectroscopy (RS), confocal laser endomicroscopy (CLE) or multiphoton laser microscopy (MPLM), are emerging for this purpose with the goal of gaining real-time histopathological information. Optical coherence tomography (OCT) has first been introduced for the detection of brain tumor tissue in 1998 by Boppart et al. (7) and has since gained increasing interest with promising potential. By measuring light interference of backscattered light from a target tissue with a reference light signal, OCT can provide real-time three-dimensional (3D) images of tissue microstructures at a spatial resolution of 5–15 μm with an imaging depth up to 3 mm in solid tissues. Low cost and non-invasiveness are further advantages of this technology. OCT provides intraoperative image impressions that are comparable to those of intraoperative ultrasound images, with the difference of an almost microscopic resolution, for the generation of these images is based on backscattered light instead of reflected sound. However, interpretation of these images is a complex undertaking. Several authors have described both qualitative (8, 9) and quantitative (10) image properties that differ in healthy and diseased brain tissue. Thus, image interpretation requires high levels of viewer expertise and its accuracy has only been reported for ex vivo imaging on small patient cohorts, varying in sensitivity and specificity in the range of 90-100% and 76-96%, respectively (9–12). Research on neurosurgical *in vivo* application, in contrast, is still mostly focused on feasibility (8, 9, 13–15). A variety of different OCT systems, e.g. time domain (TD) OCT, spectral domain (SD) OCT, etc. with different set ups, e.g. hand-held imaging probes, stationary systems, etc. have been introduced for intraoperative application. Each system in turn operates on fixed basic settings, such as specific wavelength of probing light, imaging rate and dimensions of field of view, with influence on axial and lateral image resolution. The resulting lack of comparability combined with varying data regarding accuracy of RTD and complex image interpretation are among a few of the reasons why OCT imaging of the central nervous system (CNS), in contrast to ophthalmology, has only limited acceptance in intraoperative application. Therefore, in order to assess the benefit of such a system, we propose a systematic analysis of the respective applicability and accuracy in the detection of tumorous tissue in comparison to techniques that are currently applied in clinical practice.

## 2 Material and methods

### 2.1 Optical coherence tomography

A microscope-integrated SD OCT System by Haag-Streit (OptMedt iOCT, Wedel, Germany) was used with a central imaging wavelength of 830nm at an A-scan rate of 35000/s, achieving an axial and lateral resolution of 8µm (full width half maximum (FWHM) in air) and 23µm (FWHM in air), respectively. In all scans, the working distance was set to 300 mm at zoom level 9 to achieve high resolution with sufficient relative back scattered signal intensity. The field of view was limited to a frame of 5.7x15.7, as described before (15). On average, 5 distinct locations at protocol-defined sites for representative coverage of the entire resection area were imaged after tumor tissue extraction by an experienced neurosurgeon. An additional image was taken from the surface of the tumor. Each scanning process took approximately 30 seconds to complete. By moving the microscope manually, it was aimed to achieve a 90° angle from the light source onto the underlying tissue. Thereby, an *in vivo* OCT dataset was created that consists of a total of 108 OCT volume scans of *in vivo* brain and brain tumor.

### 2.2 Specimens and histology

After imaging, MRI navigated tissue biopsies were obtained at these locations within the resection cavity in a clinical study under protocol #18-204 granted by the ethics committee of the University of Lübeck. Every tissue sample underwent histological preparation and was analyzed for validation of tissue type, residual tumor burden and cancer grade by a neuropathologist. In addition, classification and grading of the main tumor mass was performed routinely in a separate analysis. The average sample size was 4x4x2 mm. 10 sections were cut from each sample, stained with hematoxylin and eosin and segmented within a tissue labeling system. Labels consist of healthy white matter, edematous tissue, gray matter or different grades of tumorous infiltration. The distribution of the main tumor types is displayed in **Table 1**. Neuropathology found residual tumor in at least one tissue sample in 12 of the patients, where tissue samples in the remaining 9 patients were free of tumor infiltration.

**Table 1:** List of tumor entities for 21 brain tumor patients.

Patient ID	Entity	Patient ID	Entity
001	Glioblastoma	012	Metastasis (Renal cell carcinoma)
002	Anaplastic Oligodendroglioma (WHO III)	013	Metastasis (Adenocarcinoma)
003	Glioblastoma	014	Anaplastic Oligodendroglioma (WHO III)
004	Metastasis (Lymphoma)	015	Metastasis (Ovarian cancer)
005	Glioblastoma	016	Glioblastoma
006	Neuroendocrine Carcinoma (WHO III)	017	Glioblastoma
007	Glioblastoma	018	Metastasis (Melanoma)
008	Anaplastic Astrocytoma (WHO III)	019	Glioblastoma
009	Glioblastoma	020	Anaplastic Oligodendroglioma (WHO III)
010	Glioblastoma	021	Glioblastoma
011	Metastasis (Non-small-cell-lung-cancer)		

### 2.3 Image processing

In order to achieve exact correlation between tissue sample and OCT image, the field of view within the OCT volume had to be reduced to a specific region of interest (ROI), that was defined after locating the area of tissue acquisition within white light images and reproducing it within their matched OCT generated en-face images as displayed in **Figure S1** (**Supplemental Material**). The corresponding en-face OCT images were created from the original OCT volumes by using a custom-written code (Matlab 9.10.0 R2021a; The Mathworks Inc., Natick, Massachusetts). In a second step, a subvolume from within the ROI was generated that could then be used for further analysis where a comparison to the histopathological findings was possible. Each subvolume contains 40 sequential B-scans on average.

Each A-scan in a subvolume is still influenced by the roll-off and the focus function, for depth dependent signal effects have to be taken into account. Therefore, these effects need to be compensated in order to allow a sufficient analysis as has been described before by our group (16). Exemplarily, **Figure S2** shows the results of that compensation (**Supplemental Material**).

For qualitative analysis, the surface was also normalized for each OCT B-scan (**Figure S3**; **Supplemental Material**). This step simplifies visual assessment for the neurosurgeon. For quantitative and neural network analysis, patches were manually extracted from the surface normalized OCT B-scans. These patches only contain valid OCT data and no artifacts or empty information. 914 patches were extracted in total at a size of 144x56 pixel.

### 2.4 Qualitative OCT scan analysis

#### 2.4.1 Image properties

In analogy to qualitative OCT image properties for visual assessment of tumorous tissue vs. healthy brain tissue, as has been suggested by Yashin et al. (9), Böhringer et al. (8) and Yu et al. (17), five image properties that evidently indicate the underlying tissue type after tumor excision were defined. These parameters aimed to reflect common knowledge on what has been described as indicators for cancerous brain tissue alteration by using OCT imaging. In short, the idea is that myelin degradation within white matter of the brain, caused by cancerous infiltration, results in a decreased optical light attenuation in the target tissue. Also, an increase of microstructures, such as cysts, calcifications and hypervascularization within tumor tissue, that OCT imaging has shown to illustrate (17), has been described as an indicator for tumor detection. In accordance with these propositions, the mentioned parameters were combined into a visual classifier containing the following image criteria:

(1) Signal intensity (“high”/”low”) – intensity value [dB] displayed through color code, deep red signal (70 dB) with abrupt fading vs. deep blue signal (40 dB) with steady fading, average throughout the OCT subvolume
(2) Homogeneity of intensity (homogeneous/heterogeneous) – variation of the signal within the ROI OCT subvolume
(3) Penetration depth of the signal (high/low) – signal depth >500µm = high, signal depth<500µm = low
(4) Uniformity of the penetration depth (uniform/non-uniform) – varying signal depth throughout the OCT subvolume
(5) Increase in microstructures – signal shadowing within OCT image that indicates calcifications, cysts or hypervascularization

**Figure 1:**
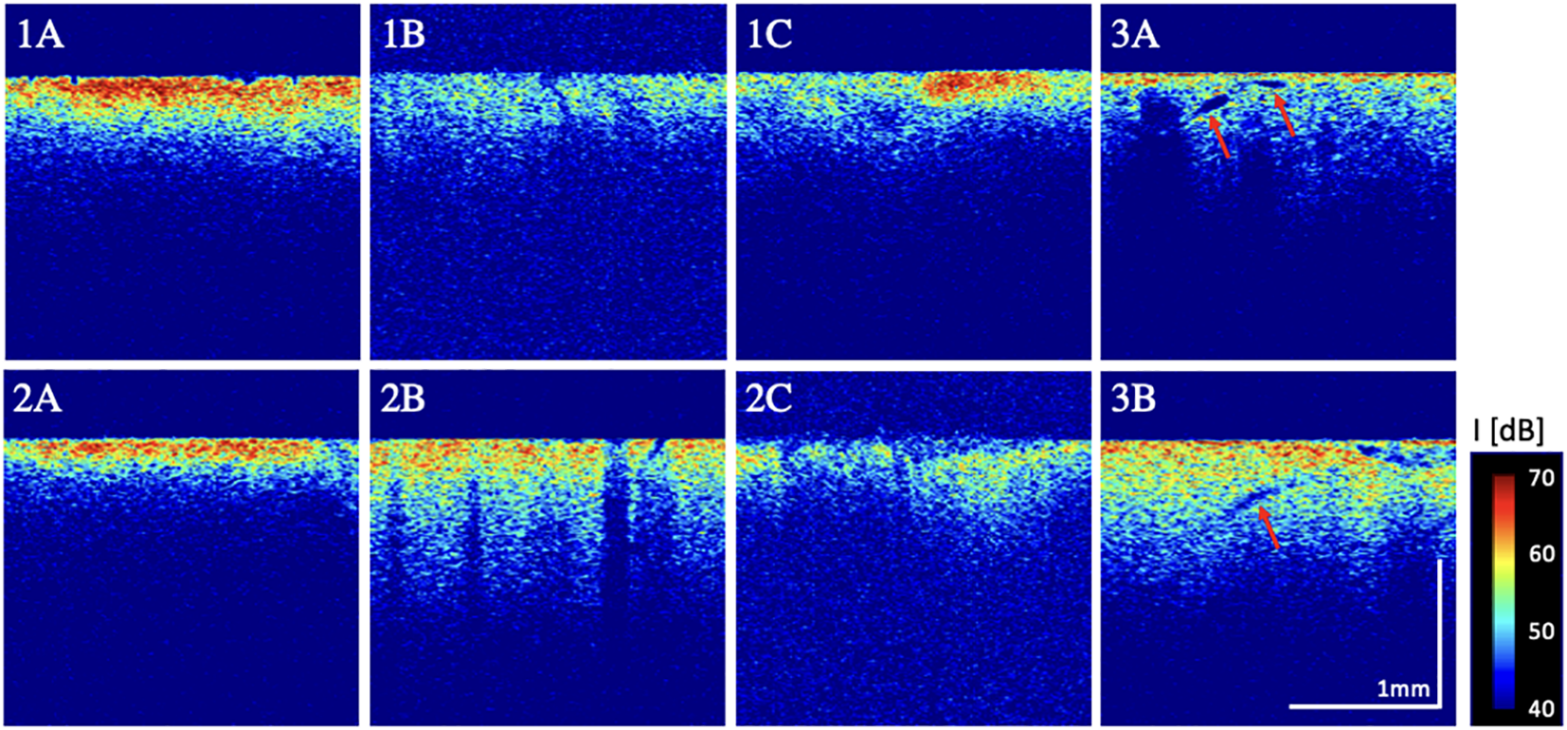
Examples of qualitative image properties. 1A: high + homogenous signal intensity, 1B: low signal intensity 1C: heterogenous intensity; 2A: low + uniform penetration depth, 2B: high penetration depth and 2C: non-uniform penetration depths; 3A/3B: increase in microstructures (here, presumably hypervascularization, pointed out through red arrows).

Analyzing OCT scans in that way aimed to take structural differences within the tissue scans into account. In analogy to surgical assessment of intraoperative ultrasound, it was thereby intended to reenact the baseline in the use of OCT imaging during tumor resection in a surgical setting. **Figure 1** exemplarily displays these image properties within OCT B-scans that were taken from the described subvolumes.

#### 2.4.2 Visual classifier

**Figure 2:**
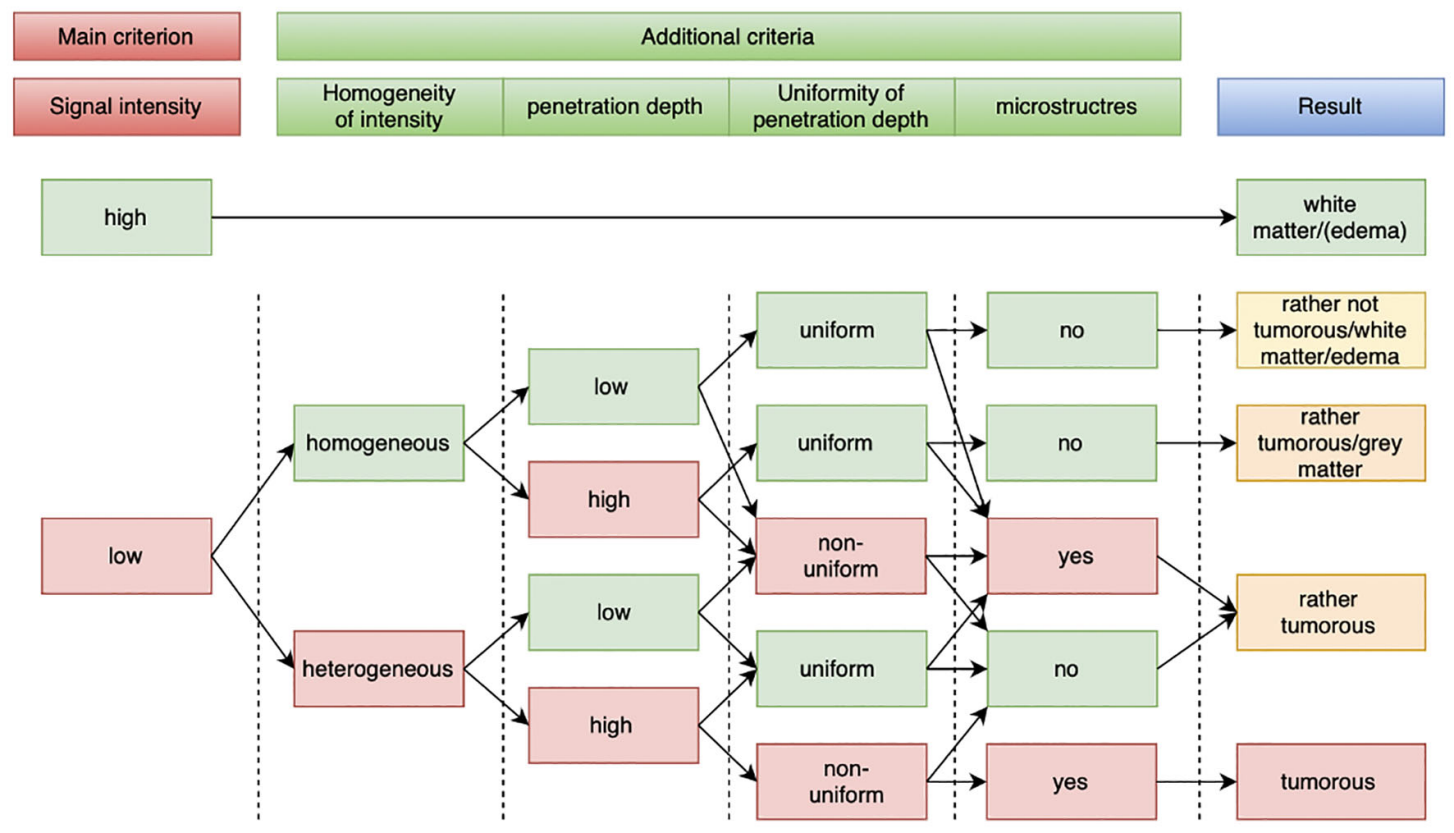
Flowchart for visual classifier. OCT signal intensity was set as main criterion. Homogeneity of signal, penetration depth, uniformity of penetration depth and the appearance of micro structures were set as additional criteria. Image properties that indicate healthy tissue are highlighted in green rectangles; image properties that indicate tumorous tissue are highlighted in red rectangles.

In correlation with our findings from ex-vivo OCT imaging and literature (9), the intensity of the signal (1) was defined as the major criterion for the differentiation between white matter and tumorous tissue. High signal intensity was therefore representative for white matter and called “*white matter*”. The additional criteria (2) – (5) were only considered when signal intensity was graded low. In this case, [(2) = homogeneous] + [(3) = low] + [(4) = uniform] + [(5) = no microstructures] was defined as “*rather not tumorous/white matter/edema*”. Whenever one or more of the additional criteria were graded otherwise, the visual classifier was set to export “*rather tumorous*”, with the exception of [(1) = low] + [(2) = homogeneous] + [(3) = high] + [(4) = uniform] + [(5) = no microstructures] which was set to export “*rather tumorous/gray matter*”. Whenever signal intensity was graded low [(1) = low] + [(2) = heterogeneous] + [(3) = high] + [(4) = non-uniform] + [(5) = microstructures], the visual classifier was set to export “*tumorous*”. For further illustration see **Figure 2**.

Visual analysis of OCT subvolumes was performed by two neurosurgeons, one surgical attending and one surgical resident, in consecutive turns and later compared regarding the concordance of the results. Both observers were shown respective OCT scans blinded to location of sample acquisition and histopathological finding and merely evaluated main and additional scan criteria. No training sets were provided. An open-source software (ImageJ, 1.53a, Wayne Rasband, National Institute of health, USA) was used for OCT image visualization and filemaker (Claris Filemaker Pro 19) for data storage and classification. It was recorded whether the used OCT scans were eligible for analysis, for some subvolumes lacked image quality, and visually evaluated the degree of concordance between white light image and OCT generated en-face image after matching. Scans with no correlation were also excluded from further analysis.

### 2.5 Quantitative OCT scan analysis

#### 2.5.1 Tumor classification using optical properties

Quantitative analysis included the determination of optical properties, which can be extracted from an OCT A-scan A²(z)’ (18–21). The resulting signal A²(z)’ contained only the information of the medium, which the incident light hit:


Eq. 1 $${\text{A}}^{2}{\left(\text{z}\right)}^{'}=\text{I}*\text{exp}\left(-2µ\text{z}\right)$$

µ is the attenuation coefficient, which describes how strong the signal decreases along the depth axis. I is the maximum backscattered intensity, which was detected by the OCT system. Both parameters are influenced by the scattering anisotropy of the imaged medium, which changes due to different tissue compositions (e.g. increasing tumor infiltration) (22, 23). In order to extract the two optical parameters from an OCT A-scan, a linear least-squares fit was applied to the logarithmized OCT A-scan. The quality of each fit was determined with the coefficient of determination $r^{2}\in \left[0,1\right]$ , which was defined as:


Eq. 2 $$r^{2}=1-\frac{\sum_{i=1}^{N}{\left(y_{i}-f_{i}\right)}^{2}}{\sum_{i=1}^{N}{\left(y_{i}-\bar{y}\right)}^{2}}$$

y defines the measured logarithmized OCT A-scan values and f (µ, I) the signal values for the specified I and µ. r² correlates with the homogeneity of the OCT A-scan. The higher r², the closer are the measured A-scan values to the determined function f (µ, I). Meaning the A-scan is more homogeneous, than an A-scan, which was fitted with and smaller r². Therefore, r² was used to evaluate structural information on signal homogeneity, while I and µ resemble optical properties of the tissue.

For the quantitative analysis all extracted OCT B-scan patches were averaged to one A-scan each. The fit was applied to a region of interest, which was 300 µm long and started 20 µm after the maximum measured intensity of the A-scan. For statistical analysis respective mean values were evaluated regarding normal distribution using the Shapiro-Wilk test. For pairwise comparison of the respective values, the Wilcoxon signed-rank test was used. For the classification based on the optical properties a support vector machine with a linear kernel was used. The training configurations were the same as for the classification using artificial intelligence (2.5.2). The optimal cost parameter for the regularization was empirically determined to be 0.1.

#### 2.5.2 Tumor classification using artificial intelligence

Two different classification approaches were applied to 914 OCT B-scan patches. The images were normalized by subtracting the mean image value and division of the standard deviation before being put into the classification. The first classification approach used the OCT B-scans directly to train a convolutional neural network (CNN) in order to identify healthy tissue from pathological brain tissue. The second approach used an autoencoder network (AE) to extract unsupervised features from the B-scan patches. The found features were then used as the basis for the classification. **Figure S4** shows an overview of the architecture used for the classifications (**Supplemental Material**).

The training of the CNN consisted of a leave-one-out approach. Each patient was once used as the test data, while the remaining patients were used for the training. The training was performed in batches, which contained 32 OCT B-scans for 100 epochs. For each training configuration the specificity, sensitivity and balanced accuracy were calculated and the overall performance of the approach was evaluated by the mean sensitivity and specificity of all training folds.

The AE consisted of an encoder and a decoder network. The output of the encoder was then used to train a fully connected neural network, which classified the data. The training and test data was randomly selected from the pool of all available OCT B-scan patches with a split ratio of 30%. The AE was trained on batches of 32 images. For the classification a fully connected neural network (FC) was used. The training procedure was the same as for the CNN.

### 2.6 Fluorescence-guided surgery

After induction of general anesthesia and before opening of the dura, 4 mg/kg body weight Fluorescein ALCON^®^ (10% with 100mg/ml; Zul.-Nr.: 6375757.00.00) was used intravenously on every patient for intraoperative fluorescence guidance. Tumor resection was performed >15 minutes after injection of FNa. Fluorescence filters were inserted into the operating microscope which allow excitation with wavelengths of 460nm to 500nm and observation with a cut-on wavelength of 510 nm. Prior to tumor resection, fluorescence imaging was used to confirm localization of the invisible tumor in relation to the brain surface. For the most part, tumor resection was performed with the filter turned off and merely turned on for locating tumor tissue and residual tumor at the resection edge (see **Figure S5C**; **Supplemental Material**). Whenever tissue samples were obtained, it was documented whether that tissue showed a fluorescent signal or not. BrainLab VectorVision (Brainlab AG, Munich, Germany) was used for neuronavigation and consulted in all cases for extend of resection. All tissue samples were neuronavigated, as can be seen in **Figure S5A and B**; **Supplemental Material**). No intraoperative ultrasound was used.

### 2.7 Early post-operative MRI

Within 24 hours after surgery, every patient received a 3D gadolinium MRI to view tumor residues. These results were subsequently compared to histopathology and analyzed for accuracy of RTD. Whenever histopathology showed residual tumor and neuroradiology confirmed GTR, that scan was classified as false negative.

### 2.8 Microscope and scanning applicability

After every surgery, the subjective appreciation of this system was recorded by interviewing each neurosurgeon with a set of questions about usability, convenience and arising issues. In detail, we aimed at documenting the individual applicability of the Haag Streit^®^ (HS Hi-R NEO 900) microscope with its integrated OCT scan technology with regards to mobility of the microscope arm, time-effectiveness of OCT scanning in relation to interruption of the intraoperative workflow and accessibility of the region of interest within the resection cavity.

## 3 Results

### 3.1 OCT scan quality

Unprocessed scans did not hold sufficient information for tissue evaluation. For this reason, on-sight analysis of real time tissue imaging was not yet feasible and tissue scans had to be post-processed after surgery as is explained in 2.3. Many artifacts were found in the final volume scans, such as signal fold-over or lack of signal. This is why only 44 volume scans from 18 patients were included in the final analysis, resulting in a 60% drop out of the gathered data.

### 3.2 Surgical appreciation of the microscope-integrated iOCT system

The overall intraoperative appreciation of this system was rated rather poor. Only in 16% of the cases did the respective surgeons rate the applicability as “good”, whereas 84% rated “rather good” or “bad”. Especially the mobility of the microscope, as well as the scanning time seemed to lead to this unappreciation. The execution of imaging within the resection cavity was also limited to the fact that a 90° angle onto the underlying tissue was not always achievable, which is why only in 43% of the cases surgeons rated “good accessibility” and the rest rated “poor” – or “very poor accessibility”.

### 3.3 Qualitative OCT scan analysis

**Figure 3:**
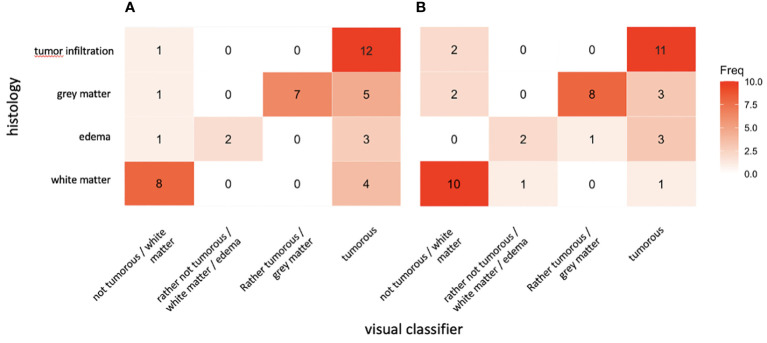
Heatmaps for degree of correlation between visual classification and histophatological finding for observer 1 **(A)** and observer 2 **(B)** from 44 subvolume scans.

**Figure 3** shows heatmaps for the correlation of histopathology and visual classification. Both maps show an upward tendency from bottom left to top right, indicating correlation between visual assessment and histopathological finding. By simply regarding the detection of white matter and tumor infiltration, sensitivity and specificity of the applied visual classifier ranged from 75% and 89% (balanced accuracy 82%) in observer 1 to 91% and 83% (balanced accuracy 87%) in observer 2, respectively.

### 3.4 Quantitative OCT scan analysis

#### 3.4.1 Tumor classification with optical properties

**Figure 4:**
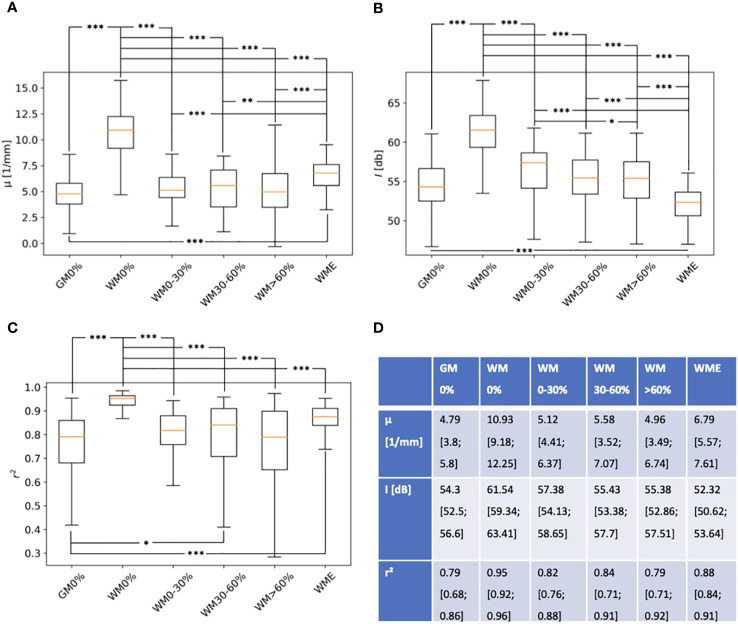
Boxplots for three extracted optical properties **(A)** - μ, **(B)** – I and **(C)** – r^2^ in regard to different tissue types (GM0%, gray matter with 0 tumor infiltration; WM0%, white matter with 0% tumor infiltration; WMO-30%, white matter with 0-30% tumor infiltration; WM30-60%, white matter with 30-60% tumor infiltration; WM>60%, white matter with > 60% tumor infiltration and WME, white matter with edema). Highly significant differences (p-value <0,001) were marked with ***, significant differences (p-value < 0,01) were marked with **, significant differences (p-value < 0,05) were marked with *. Numerical values of the optical properties are displayed in **(D)**, representing median, 25 and 75 percentiles.

**Figure 4** displays the results for the determination of the three optical properties through a linear least-squares fitting approach. The results show that all parameters decrease with increasing grade of tumor infiltration. The measured values suggest that there is a significant difference in the determined optical values of healthy white matter and tumor infiltrated white matter. The measured values for healthy white matter indicate, that the tissue is a smooth tissue with high scattering properties, which is why the attenuation and the backscattered intensity are high. Tumor infiltrated white matter on the other hand has the opposite properties. The r²-value indicates a more heterogeneous structure with lower scattering properties. Gray matter shows similar optical properties to the tumor infiltrated white matter. The edematous tissue shows a more homogeneous tissue structure than tumor infiltrated white matter but is more heterogeneous than healthy white matter. Regarding the attenuation and the backscattered intensity, edema is closer to tumor infiltrated white matter, than healthy white matter.

**Figure 5:**
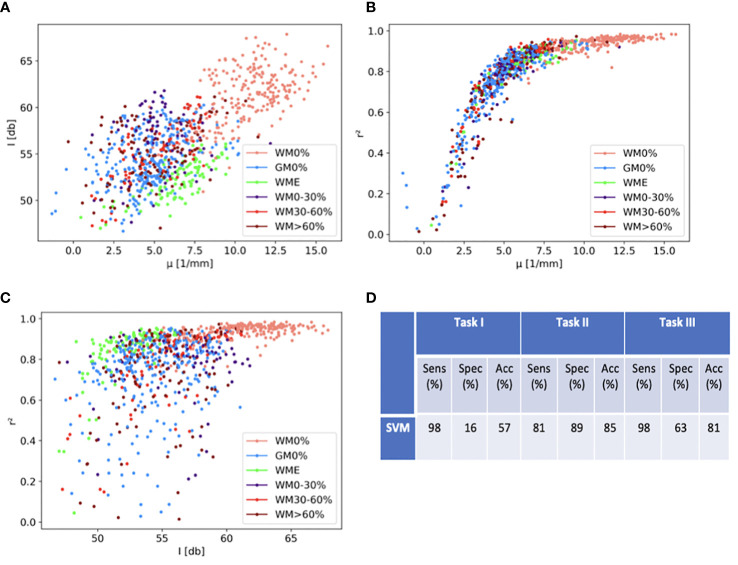
Scatter plots of extracted optical properties in regard to tissue labels (GM0%, gray matter with 0 tumor infiltration; WM0%, white matter with 0% tumor infiltration; WM0-30%, white matter with 0-30% tumor infiltration; WM30-60%, white matter with 30-60% tumor infiltration; WM>60%, white matter with >60% tumor infiltration and WME, white matter with edema). **(A)** shows backscatter intensity against attenuation coefficient, **(B)** displays attenuation coefficient against fitted measured r2, **(C)** shows backscatter intensity against fitted measured r2. **(D)** shows results for sensitivity (Sens), specificity (Spec) and balanced accuracy (Acc) for specific classification tasks with support vector machine (SVM) classification. Task I includes full data, Task II includes classification of tumor infiltration in white matter (excluding GM0% and WME) and Task III includes classification of highest tumor infiltration grade in white matter (excluding GM0%, WME, WM0-30% and WM30-60%).

**Figure 5** shows relations between optical properties separately by displaying measured optical properties for each OCT B-scan patch. Values for white matter and different grades of tumor infiltration were separated in order to better visualize forming clusters. The measured similarity between tumor infiltration and gray matter creates a big cluster. The measured values for white matter and edematous tissue seem to create a cluster of their own. In order to assess the accuracy of tumor detection through consulting these image property clusters, a support vector machine (SVM) was used in a combined approach to create binary linear categories for the calculation of sensitivity and specificity. For the classification, three different classification tasks were defined. The first task (I) uses the full data available to the classification. Here healthy gray and white matter were assigned to the non-pathological class, while the other tissue labels were assigned to the pathological class. The second task (II) only focuses on the separation of white matter with 0% tumor infiltration and white matter with tumor infiltration. The third task (III) only includes healthy white matter and white matter with >60% tumor infiltration. **Figure 5D** displays sensitivity, specificity and balanced accuracy values for each task. The data show high accuracy (85%) for the separation of white matter from all degrees of tumor infiltration (tasks II). However, including gray matter and edematous tissue into the non-pathological class compromises accuracy (57%) in that separation task (task I).

#### 3.4.2 Tumor classification with artificial intelligence

The two classification approaches were trained as explained in section 2.5.2, calculating the mean sensitivity and specificity over all test configurations, as explained in 3.2.1. The results for all configurations are displayed in **Table 2**. The data shows that both approaches achieved good classification results for the classification of white matter from the different grades of tumor infiltration. On the contrary, they struggled to achieve good results for the classification of the healthy tissue from the pathological tissue (task I). Overall, the AE+FC approach showed the better performance.

**Table 2:** Sensitivity (Sens), specificity (Spec) and balanced accuracy (ACC) for both CNN and AE + FC classification approaches for specific classification tasks. Task I includes full data, Task II includes classification of tumor infiltration in white matter and Task III includes classification of highest tumor infiltration grade in white matter.

Classification approach	Task I			Task II			Task III		
	Sens (%)	Spec (%)	Acc (%)	Sens (%)	Spec (%)	Acc (%)	Sens (%)	Spec (%)	Acc (%)
CNN	59	77	68	84	79	82	87	79	83
AE + FC	65	66	66	86	83	85	90	84	87

### 3.5 Fluorescence guidance

In 16 patients, the operating neurosurgeon agreed on having achieved GTR by assessing the lack of fluorescence signal and consulting neuronavigation. In the 5 remaining patients, the operating surgeon expressed the suspicion of residual tumor. In 9 cases, there was no sign of tumor infiltration within the tissue samples, when the neurosurgeon called for GTR. However, neuropathology showed tumor infiltration in at least one tissue sample in the remaining 7 patients, when the neurosurgeon called for GTR. All of the 5 patients, where the neurosurgeon expressed concerns for subtotal resection, showed at least one tissue sample with tumor infiltration. Therefore, by merely assessing fluorescence signaling and consulting neuronavigation, only 67% of the expected results where rightfully reflected through histopathological findings, with a sensitivity of just 42%. **Table S1** lists the number of cases with true negative/positive and false negative/positive results (**Supplemental Material**).

### 3.6 Early post-operative magnetic resonance imaging

In 12 patients, neuroradiology did not show gadolinium enhancement in the early post-operative MRI, while free margins were only histopathologically detected in 9 patients. 3 patients were therefore falsely found tumor-free through neuroradiology, all of which were glioblastoma cases. In the remaining 9 patients, where MRI showed signs of residual tumor, all at least showed one tissue sample with tumor infiltration. The estimated early MRI based sensitivity in this study for the detection of residual tumor was 75%.

## 4 Discussion

We aimed to analyze a microscope integrated OCT system (iOCT by Haag-Streit) with the intention of gaining insight into intraoperative *in vivo* applicability and accuracy in the detection of residual tumor in a range of approaches and compare these findings to other systems and techniques that are commonly intraoperatively used for this purpose. Miniaturizing large imaging instruments for clinical *in vivo* use is a complicated challenge, where integration into the surgical workflow needs to be feasible without much effort. Therefore, integrating such a system into a surgical microscope appears to be an elegant way of overcoming that obstacle. On-sight interpretation of what is illustrated, however, still remains a difficulty. Hartmann et al. have shown the benefit of *in vivo* OCT for the visualization of distinctive anatomical targets, such as the subarachnoid space (24), vascular anomalies (25) or arachnoid cysts (26), achieving high image quality. In contrast, differentiation of infiltrating tissue, in which a morphological transition is not apparent to the human eye, is a more complex undertaking. Analysis of qualitative and quantitative image properties on ex vivo tissue have well been described as targets for tissue distinction. In this work, it was proven that these approaches were reproduceable in an *in vivo* set up with high values for overall accuracy and demonstrated that machine learning has promising potential for this purpose in a microscope-integrated system.

Introducing new tools in the OR is always a challenge. This might be one of the reasons, why the overall appreciation of this system might not yet be satisfactory. A major challenge is optimal manual adjustment of the microscope for the exploration of the resection cavity where different dimensions of resection cavities led to an inconvenient accessibility. Focusing at a 90° angle on the surface of the tissue was described as particularly difficult. The integration of a robotic system that would automatically traverse resection cavities would simplify this step. Technical developments in this area are on the rise and have well been demonstrated experimentally (27). This, combined with a lack of intraoperative scan quality validation, led to a large number of scans with artifacts that had to be excluded from further analysis. However, finding the right resection edge with current standard techniques is equally challenging in brain tumor surgery today.

Even though authors from the multicentric prospective phase II study (FLUOGLIO) reported a sensitivity and specificity of FNa guidance in identifying tumor tissue with 80.8% and 79.1%, respectively (28), in this study a sensitivity of only 42% was obtained. This might be explained by the different set up of the study design, as well as a more representative cohort size in the phase II study. In this regard, only a 75% sensitivity for early post operative MRI was found in this work, which in analogy and in comparison to other research groups seems rather low. Heßelmann et al. reportet a 95% sensitivity for RTD using intraoperative MRI (29). Nevertheless, these findings show that current techniques for intraoperative RTD are not always easy applicable and far from reliably accurate.

The iOCT system provides real-time dynamic feedback of the underlying tissue, which in its form reminds the viewer of an ultrasound similar signaling. Yashin et al. and Yu et al. proposed a qualitative analysis in regard to specific signal features that could be applied intraoperatively by a trained neurosurgeon. For this reason, a visual classifier was installed that would provide the viewer with a simple step-by-step interpretation tool for a systematic decision-making basis. The respective authors demonstrated qualitative differences in image properties using an ex vivo OCT imaging probe on healthy and diseased brain tissues, whereas in this study a contactless *in vivo* approach was carried out. Yet, through a defined scan distance sufficient relative back scattered signaling was generated, which in turn made it feasible to achieve comparability throughout the cohort. It is noteworthy to say that real-time image analysis on sight was not feasible, for these images had to undergo post-processing, as is described in 2.3, to make them visually distinctive.

In a blinded retrospective analysis of *in vivo* OCT scans, a sufficient correlation of diagnosed tissue type and histopathology was found. Differentiation of gray matter from tumor infiltrated tissue deemed to be of greater difficulty, for signal intensity is low in both tissues due to less light scattering. Here, additional criteria were not always sufficient to differentiate between the two, which is why values of accuracy for both observers ranged moderately, with a somewhat significant interobserver variability. However, both observers were well able to correctly classify healthy white matter from tumor infiltrated tissue through visual assessment. In this simple approach it was demonstrated that just by visually assessing *in vivo* OCT signaling, one attending neurosurgeon and one resident in neurosurgery were able to differentiate tissue with ranging values for balanced accuracy from 82-87%, which is significantly higher when compared to what could be demonstrated for FNa guidance and neuronavigation in this study and similar to higher from what authors from FLUOGLIO found. Applying real-time image processing onto *in vivo* OCT images, could therefore be a valuable add-on for current intraoperative tissue distinction.

The results for measured optical properties also confirmed findings of other research groups. Highly significant differences in all three properties for the respective tissue labels were found, especially in the comparison between healthy white matter and the different degrees of tumor infiltrated tissue with p values far below a set limit value of 0.05. Looking at the attenuation coefficient, a direct comparison to values from other research groups and the presented values is not sufficient, for light scattering increases with decreasing imaging wavelength (30). Most groups used an imaging wavelength of 1300 nm, whereas the iOCT System functions on 850 nm. However, relative relations of the attenuation coefficient can be compared (8, 10, 14, 18, 31). Furthermore, overall trends match reports from other groups. Yashin et al. explained high light attenuation of healthy white matter with the presence of highly scattering myelin fibers, which for the most part are not present in healthy gray matter. In theory, the higher the degree of tumor infiltration the higher the degradation of myelin fiber, consequently leading to a decrease of light attenuation (18, 32). For edema, Rodriguez et al. reported that edema in gray matter of mice can reduce the attenuation coefficient by up to 8% (31). In this work, the attenuation coefficient of edema in white matter was around 40% smaller than in healthy white matter. These differences may be explained with a different initial set up for both experiments, but the general trend is the same. The relative differences are closer, when comparing the determined values of healthy gray and white matter with other research groups. For Yashin et al., the reported attenuation coefficient of gray matter was 40% smaller than healthy white matter (18). For Kut et al. the difference was 55% and for Almasian et al. 43% (10, 14). For values reported in this work, gray matter was 56% lower than healthy white matter. Unlike other research groups, this work focused on differentiating tissue at the resection edge, where tissue with different degrees of tumor infiltration could be assessed. This complicates comparison to other groups, since most groups do not differentiate various stages of tumor infiltration in the detail this works does. Kut et al. provided a mean attenuation coefficient for tumor infiltrated white matter (3.5 ± 0.8 1/mm) and tumor core (3.9 ± 1.6 1/mm). The relative difference to healthy white matter is similar to the difference, which can be derived from the values from this work. While backscattered intensity showed similar behavior to the attenuation coefficient within different tissue labels, relative differences were much higher. This is a similar observation to findings of Venkata et al., who found that in confocal microscopy, measured reflectivity changed stronger than the scattering coefficient if anisotropy of a medium changes (23). In the case of this work, the anisotropy of brain tissue changes for example with the degree of tumor infiltration.

Structural analysis concerning the r²-value was very rudimental compared to the analysis of other research groups (14, 33, 34). The value correlated well for white matter and different stages of tumor infiltration. The r²-value decreased with increasing tumor infiltration, for homogenous structure of white matter is disturbed by upcoming cysts, hemorrhage or vessel proliferations (9). Lenz et al. showed that healthy gray matter is a homogenous tissue, comparable to healthy white matter, which stays in contrast to the presented r²-values (33). The reasons for the differences could be that in some cases, surface detection in scans from cortex tissue falsely detected arachnoid mater, which led to an unbalanced normalization of the actual tissue and thus to an uneven alignment. This could also be the reason, why it was more difficult to correctly classify gray matter from tumor infiltrated tissue in the qualitative approach, for additional qualitative criteria were less precisely to assess.

When combining all optical properties for classification with the help of a support vector machine, the best results for specificity was assessed for task II in comparison to all of the approaches that are displayed in this work with a value of 89%. The respective sensitivity for this task was 81% with a balanced accuracy of 85%, which is superior in accuracy in comparison to FNa guidance found both in literature and this work.

The classification based on neural networks also achieved superior results for the separation of white matter and tumor infiltrated tissue with the highest sensitivity for the AE + FC approach in task II (Sensitivity and Specificity of 86% and 83%, respectively). These results are comparable to what has been described for 5-ALA guided surgery, where mean sensitivity and specificity in distinguishing tumor from healthy brain tissue at the resection edge ranged between 83 and 87% and 89 and 91%, respectively, in multiple meta-analyses (35). The results on *in vivo* data for this separation task, displayed in this work, even hold up with results achieved on ex vivo data with OCT systems with better resolution. Gesperger et al. achieved a specificity of 100% and a sensitivity of 93% on ex vivo brain data, which was acquired with an optical coherence microscope system with a lateral resolution of 1.8 µm and an axial resolution of 0.88 µm (36). Juarez-Chambi et al. achieved sensitivity of 99% and a specificity of 86% with an A-scan based approach (11). The data consisted of ex vivo OCT A-scans acquired by an OCT system with a lateral resolution of 16 µm and an axial resolution of 6.4 µm. However, all approaches struggled, when it came to the classification of healthy tissue and pathological tissue. Gray matter provided for extremely similar OCT scans when compared to tumor infiltrated tissue in this work, as has been described in the qualitative analysis approach, which is why task III does not present sufficient results for the classification based on artificial intelligence either.

When assessing the utility of OCT technology, it is evident to consider other laser-based imaging modalities such as RS, MPSM or CLE, that have also been investigated for intraoperative tissue differentiation. Having shown promising results for intraoperative brain tumor detection in multiple study designs with ranging sensitivity and specificity values of 90-96% and 94-100%, respectively (37–39), all share disadvantages of requiring either tissue removal from the surgical site for ex vivo application or requiring a contact-based imaging probe *in vivo*, which is not necessary in the technology presented in this work. In the case of CLE for example, *in vivo* optical biopsies are obtained using fluorescent light reflection of tissue at question by a hand-held probe in a contact-based manner and are simultaneously examined by a neuropathologist, that was specifically trained to interpret CLE images, at a distant cloud-based workstation. OCT, on the other hand, allows for non-contact *in vivo* application without the need of further imaging equipment and the near real-time prospect of automated tissue classification.

## 5 Conclusion

Due to still inconvenient surgical application and the need of image post-processing, the use of this iOCT system has not yet shown to improve the intraoperative decision-making process concerning the extent of tumor resection. However, qualitative and quantitative *in vivo* OCT data analysis has proven to contain additional information on residual tumor, supporting what has well been described for ex vivo OCT brain tumor scanning. More than half of the scans did not seem fit for further analysis, which for the most part was caused by a missing intraoperative scan quality validation. In the remaining scans, *in vivo* OCT scanning provided for higher values of accuracy in RTD in direct comparison to FNa guidance or early post operative MRI in this study, indicating the possibility of providing complementary information on tissue at question at the resection edge. In particular, the use of artificial intelligence for image feature recognition has shown the most promising results and might be crucial to achieve high accuracy in RTD, expanding current intraoperative methods and even exceeding them in accuracy, while not yet in applicability.

## 6 Outlook

OCT technology integrated into a surgical microscope is a system in evolution. With further development in user-friendly application and integration of real time tissue analysis, this system has high potential for future intraoperative use for RTD. In an independent development step, the standard Haag Streit^®^ microscope was equipped with a MHz OCT system (40) with the ability of projecting real-time OCT data from underlying tissue as a template into the field of view of the surgeon. Applying classification methods with data that derives from this work could then enhance the extent of brain tumor excision. Currently, we aim to gather more data for the training of neuronal networks to augment classification. Subsequently, this classifier will be used for real time mapping of the operating field in a prospective approach.

## Data availability statement

The original contributions presented in the study are included in the article/**Supplementary Material**. Further inquiries can be directed to the corresponding author.

## Ethics statement

The studies involving human participants were reviewed and approved by Universität zu Lübeck. The patients/participants provided their written informed consent to participate in this study.

## Author contributions

PK: Conceptualization, formal analysis, investigation, visualization, methodology, data curation, writing original draft. PS: Contributed equally to PK, formal analysis, investigation, software, visualization, methodology, data acquisition, editing. BL: Review and editing, methodology. SS-H: Data acquisition and curation. WD: Software. CH: Histology. DT-K: Review and editing. RB: Review and editing. RH: Supervision. VT: Supervision. MB: Project administration, funding acquisition, review and editing. All authors contributed to the article and approved the submitted version.
