# Supplementary figures and images for: The neurosurgical benefit of contactless in vivo optical coherence tomography regarding residual tumor detection: A clinical study
Source: Front Oncol. 2023 Apr 13;13:1151149. doi: 10.3389/fonc.2023.1151149 (PMC10150702; doi:10.3389/fonc.2023.1151149)

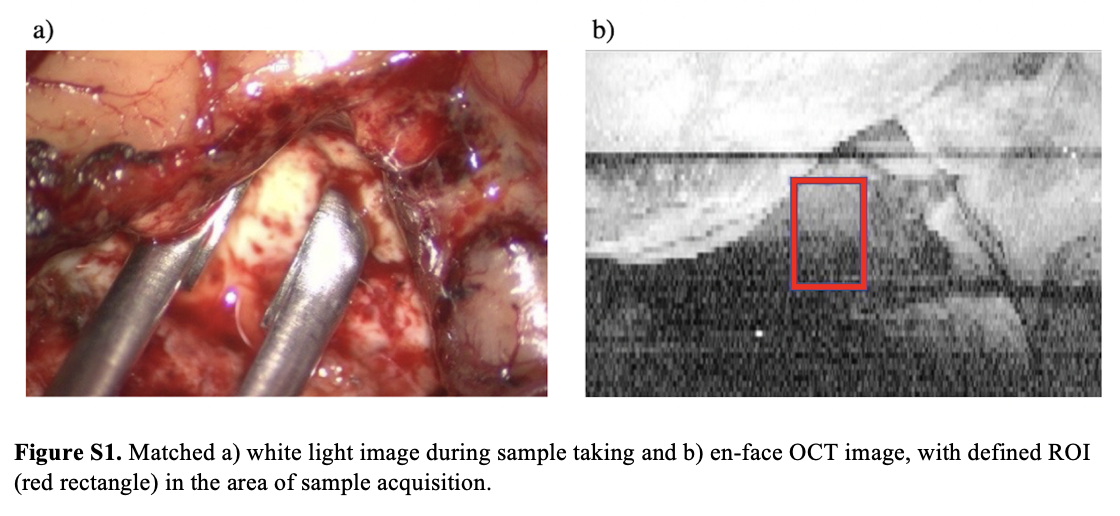

Supplement: Supplementary file 1 [file Image_1.jpg]

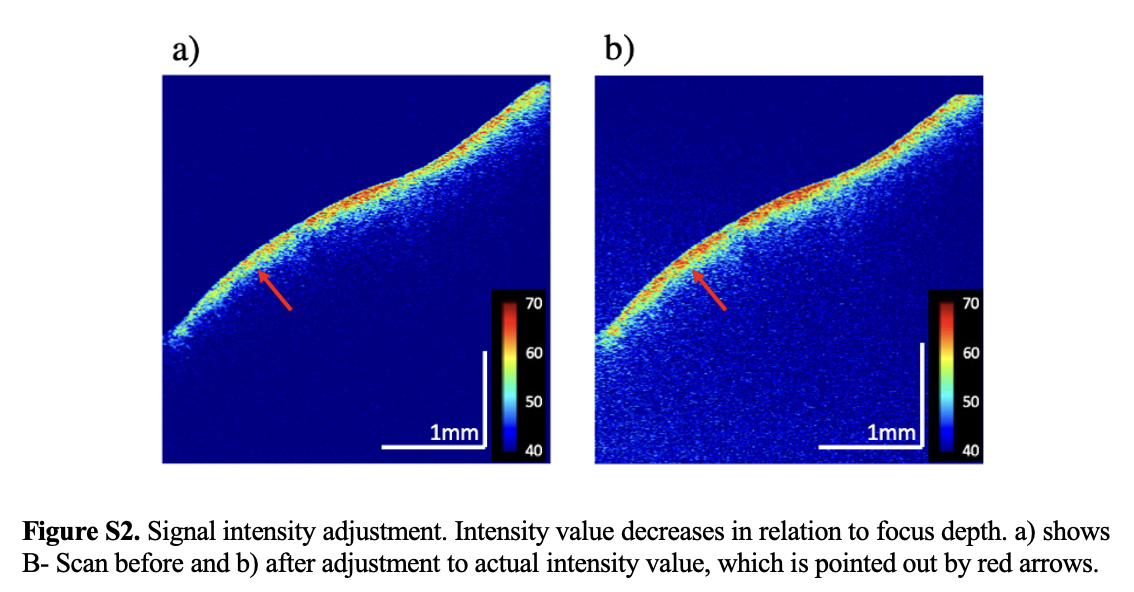

Supplement: Supplementary file 2 [file Image_2.jpg]

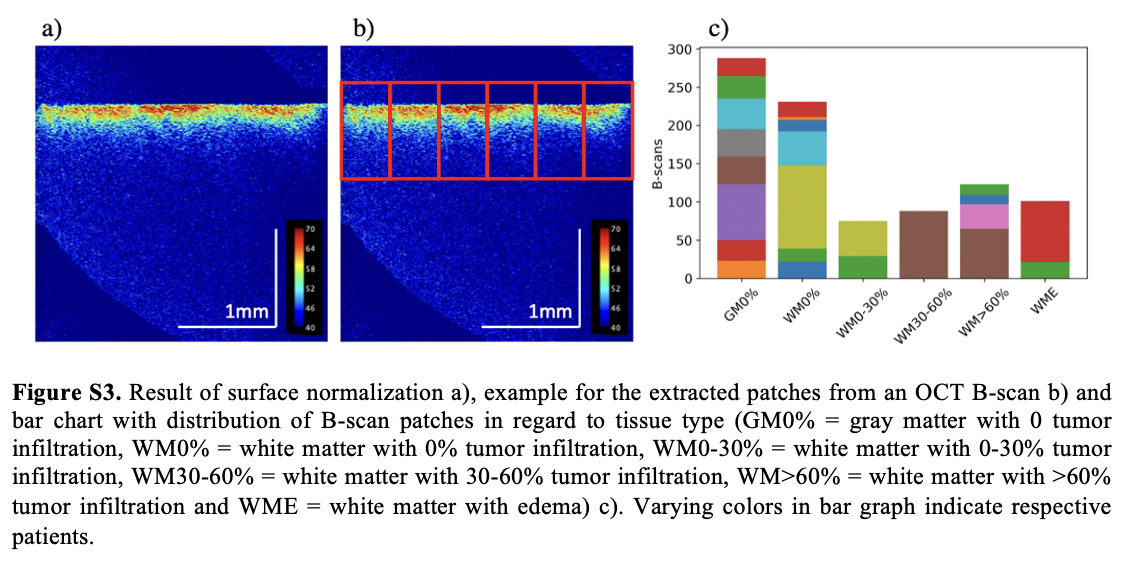

Supplement: Supplementary file 3 [file Image_3.jpg]

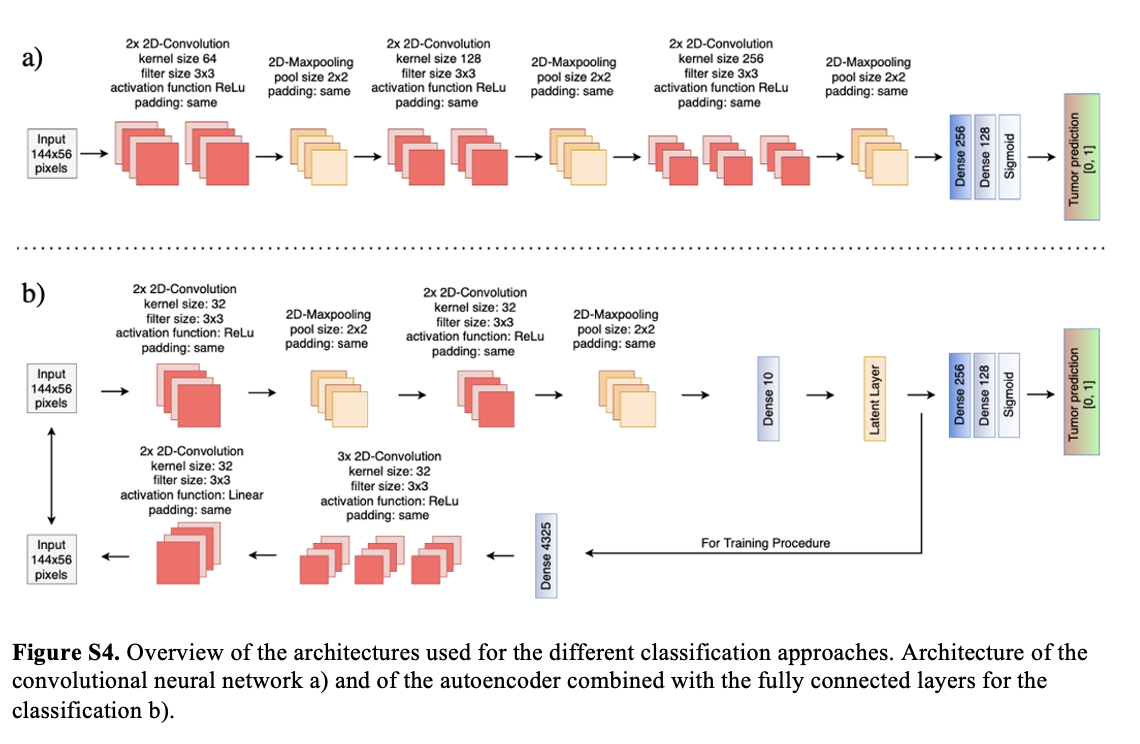

Supplement: Supplementary file 4 [file Image_4.jpg]

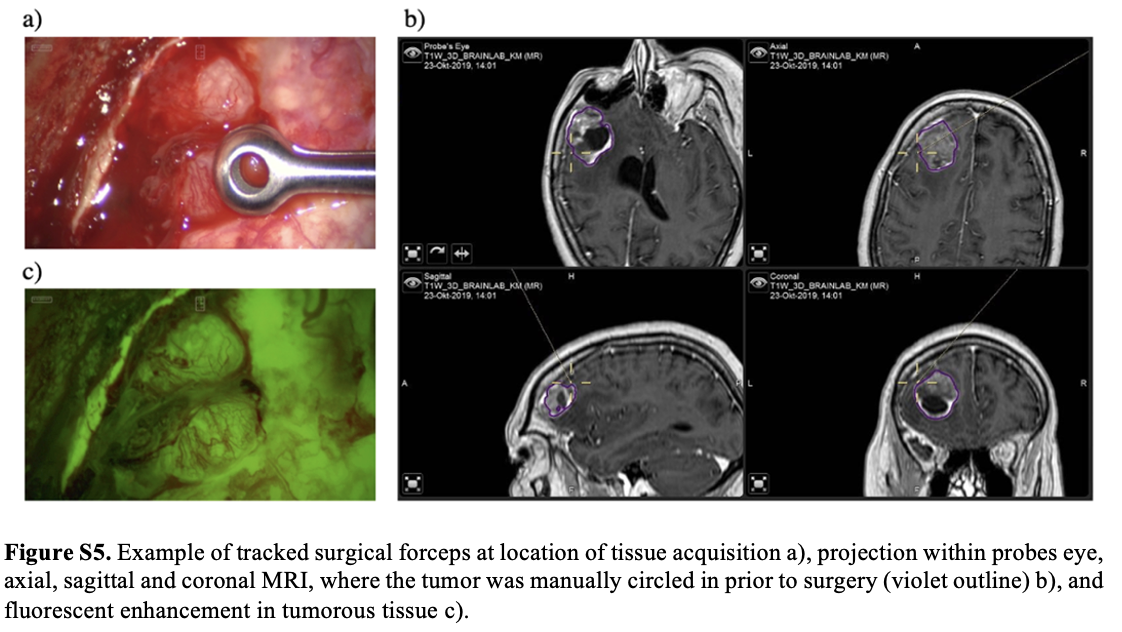

Supplement: Supplementary file 5 [file Image_5.jpg]

**Table S1**


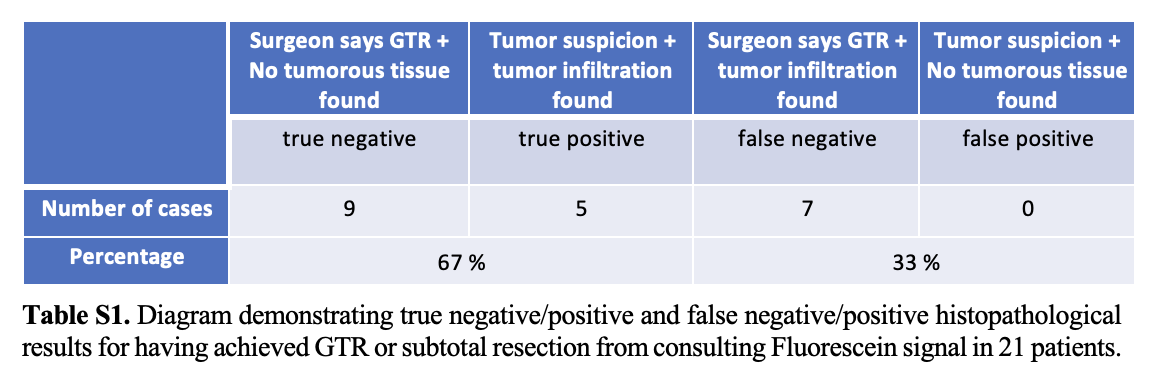

Supplement: Supplementary file 6 [file Table_1.docx]
